# Supplementary material for: Transcriptome-Wide Identification and Expression Analysis of bHLH Family Genes in Iris domestica under Drought and Cu Stress
Source: Int J Mol Sci. 2024 Feb 1;25(3):1773. doi: 10.3390/ijms25031773 (PMC10855607; doi:10.3390/ijms25031773)
Supplement: Supplementary file 1 [file ijms-25-01773-s001.zip › Supplementary Material Description.pdf]

Supplementary Material Description

Table S1. Gene cloning primers and homologous recombinant primers

Table S2. Information on 39 and *IdbHLH* genes is shown in the table including Number of Amino Acid, Molecular Weight, Theoretical pI, Instability Index, Aliphatic Index, and Grand Average of Hydropathicity.

Table S3. The prediction results of Wolf PSORT software.
