# Supplementary material for: Transcriptome-Wide Identification and Expression Analysis of bHLH Family Genes in Iris domestica under Drought and Cu Stress
Source: Int J Mol Sci. 2024 Feb 1;25(3):1773. doi: 10.3390/ijms25031773 (PMC10855607; doi:10.3390/ijms25031773)
Supplement: Supplementary file 1 [file ijms-25-01773-s001.zip › Table S1.pdf]

Table S1 Gene cloning primers and homologous recombinant primers

Gene	Primer sequences 5'→3'	Purpose
IdbHLH06	F: ATGGATCCGAACCCGATTTCGG	cloing
	R: TCAAACCTTCAGTTCTTTGATTG	
	F: tgcatgcctgcaggtcgaATGGATCCGAACCCGATTTCGG	recombination
	R: gctcctcgcccttgctcaccatggatccAACCTTCAGTTCTTTGATTG	
